# Antimicrobial resistance.

**DOI:** 10.3201/eid0707.010727

**Published:** 2001

**Authors:** T Naimi, P Ringwald, R Besser, S Thompson

**Affiliations:** Minnesota Department of Health, Minneapolis, Minnesota, USA.


### Antimicrobial Resistance

**Timothy Naimi,* Pascal Ringwald,† Richard Besser,‡ and Sharon Thompson§**
*Minnesota Department of Health, Minneapolis, Minnesota, USA; †World Health Organization, Geneva, Switzerland; ‡Centers for Disease Control and Prevention, Atlanta, Georgia, USA; and §Food and Drug Administration, Rockville, Maryland, USA

This symposium highlights selected topics in antimicrobial resistance: community-acquired methicillin-resistant *Staphylococcus aureus*, malaria, reducing inappropriate antimicrobial drug prescribing for outpatient respiratory infections, and addressing the human health impact of antimicrobial drug use in food animals.

Dr. Timothy Naimi discussed community-acquired methicillin-resistant *Staphylococcus aureus* (CMRSA) infections, which have been reported in the Western Pacific and North America with increasing frequency. Few incidence data are available, but reports show a disproportionate distribution among racial minorities and groups with low socioeconomic status. CMRSA infections resembled community-acquired methicillin-susceptible *S. aureus* infections more than MRSA infections acquired in health-care facilities.

Patients with CMRSA were young, healthy, and lacked risk factors for MRSA (recent hospitalization, dialysis, and injection drug use), and they had predominantly skin and soft tissue infections; four deaths due to invasive CMRSA infections were reported in the United States. Unlike nosocomial MRSA, CMRSA is sensitive to multiple non-beta-lactam antibiotics. CMRSA isolates have the mec A gene and distinct pulsed-field-gel electrophoresis patterns. CMRSA presents a clinical challenge because most community-acquired *S. aureus* infections are diagnosed without taking a culture, and the patient is treated with beta-lactam drugs. Improved surveillance and efforts to define risk factors are under way.

Dr. Pascal Ringwald reviewed antimalarial drug resistance, which greatly hinders malaria control since no vaccine will be available in the near future. Resistance of *Plasmodium falciparum* to chloroquine, the most frequently used antimalarial drug, is found in nearly all malaria-endemic countries. Resistance also affects all other antimalarial drugs; cross-resistance occurs against drugs in the same group.

In recent years, chloroquine-resistant *P. vivax* has been reported in Southeast Asia and in South America. Drug resistance increases illness and death, especially among children. As the number of new antimalarial agents is limited, the use of drug combinations is under evaluation. WHO supports national malaria control programs to monitor antimalarial drug resistance in sentinel sites. Decisions to change drug policy should be based on the results of monitoring in these sites. The establishment of a new policy must entail a rational use of drugs and improved compliance.

Dr. Richard Besser discussed efforts to reduce inappropriate antibiotic prescribing for outpatient respiratory infections in the United States. Over 40% of outpatient antibiotic prescriptions are for viral illnesses, which are not affected by antibiotics. CDC's educational campaign, targeted to clinicians and patients, involves partnerships with health departments, health-care delivery organizations, health-care purchasers, medical societies, and others. Materials and information are available at www.cdc.gov/drugresistance. Controlled trials of interventions demonstrated notable reductions in antibiotic prescriptions written in Denver, central Wisconsin, and rural Alaska.

Successful interventions were multifaceted and directed at both clinicians and patients. Clinician interventions included peer education using opinion leaders, improving diagnosis of acute otitis media, and feedback on group-prescribing practice. Patient interventions included education in the community (day care centers, health fairs), homes (reminder refrigerator magnets), and physician offices (videos in waiting rooms). CDC is expanding this campaign and seeking to assess its impact in larger areas.

Dr. Sharon Thompson described Food and Drug Administration (FDA) initiatives to address the human health impact of antimicrobial drug use in food animals. These initiatives include regulatory changes, antimicrobial resistance monitoring, risk assessment, promoting judicious drug use, and research. FDA has concluded that past regulations are no longer adequate to preserve important antimicrobial drugs for human use when their use in food animals may contribute to antimicrobial resistance. A proposed framework would classify antimicrobial agents according to their importance in human medicine and the likelihood that human exposure to them from consuming food animals containing them would create more resistant bacteria. Preapproval studies would assess resistance development and pathogen load in treated animals. Specified thresholds of resistance in human and animal isolates would provide risk management tools—as these thresholds were approached, drug use in animals could be restricted.

FDA is currently finalizing a risk assessment of fluoroquinolone-resistant *Campylobacter* infection and its relationship to fluoroquinolone use in poultry and is beginning an assessment of quinupristin/dalfopristin-resistant *Enterococcus faecium* and its relationship to virginiamycin use as a growth promotant. Visit the FDA website at www.fda.gov.

Address for correspondence: David Bell, Centers for Disease Control and Prevention, 1600 Clifton Road, Mailstop C12, Atlanta, Georgia 30333, USA; fax: 404-639-4197; e-mail: dbell@cdc.gov

